# Balanced DNA interpolation improves learning of genetic distance-informed embeddings in plants

**DOI:** 10.1371/journal.pcbi.1014722

**Published:** 2026-09-03

**Authors:** Lara M. Kösters, Kevin Karbstein, Ladislav Hodač, Laura Albreht, Elvira Sahuquillo Balbuena, Daniel Botello, Olivier Hardy, Phebian Odufuwa, Eva Pardo Otero, Aireen Phang, Manuel Pimentel, Rosalía Piñeiro, James Smith, Peter Wilkie, Patrick Mäder, Jana Wäldchen

**Affiliations:** 1 Biodiversity, Ecosystems and Society Group, Max Planck Institute for Biogeochemistry, Jena, Germany; 2 Data-intensive Systems and Visualization (dAI.SY), Technische Universität Ilmenau, Ilmenau, Germany; 3 Laboratory of Molecular Systematics and Evolutionary Genetics, Florida Museum of Natural History, University of Florida, Gainesville, Florida, United States of America; 4 Professorship of Integrative Plant Taxonomy, Friedrich Schiller University Jena, Jena, Germany; 5 Unit of Evolutionary Biology and Ecology CP160/12, Université Libre de Bruxelles, Brussels, Belgium; 6 Evolutionary Biology Research Group (GIBE), Departamento de Bioloxía, Facultade de Ciencias, Universidade da Coruña, A Coruña, Spain; 7 Department of Biological Sciences, Boise State University, Boise, Idaho, United States of America; 8 Evolutionary Biology Research Group (GIBE), Centre of Information and Communication Technologies (CITIC), Universidade da Coruña, A Coruña, Spain; 9 Singapore Botanic Gardens, National Parks Board, Singapore, Singapore; 10 Taxonomy and Macroecology Group, Royal Botanic Garden Edinburgh, Edinburgh, United Kingdom; 11 Evolutionary Biology Research Group (GIBE), Interdisciplinary Centre of Chemistry and Biology (CICA), Universidade da Coruña, A Coruña, Spain; 12 Institute of Biodiversity, Ecology and Evolution, Friedrich Schiller University Jena, Jena, Germany; 13 German Centre for Integrative Biodiversity Research (iDiv) Halle-Jena-Leipzig, Leipzig, Germany; Xiamen University, CHINA

## Abstract

In taxonomic research, traditional phylogenetic tree and structure analyses of genetic data are increasingly complemented by machine-learning-based identification and representation learning. Although the amount of DNA data needed to train state-of-the-art machine learning models often exceeds what can realistically be collected and sequenced in biological studies, the number of samples can be extended artificially through data augmentation. Genetic data augmentation usually refers to the introduction of random base variations, translocations, and reverse complementing. These augmentations do not take into account the inherent structures of populations and species, potentially blurring the lines between entities within genetic datasets. Here, we propose DNAInterpolator, an approach based on interpolation of DNA sequences within a given dataset that presents a neighbor-guided alternative to random mutations. We tested interpolation as an augmentation technique using four flowering plant datasets and an artificial neural network trained to predict genetic distances between paired samples. To address unequally distributed distances within our training datasets, we examined the effect of balancing the distance distribution by curating interpolated sequences. We found that balancing helps models capture genetic distances across the full distance range by strengthening performance in underrepresented regions of the distribution. Our new approach leverages the potential of taxonomic DNA datasets for modern machine learning applications.

## Author summary

In recent years, interest in the study of genetic data using machine-learning-based approaches is increasing. However, for research focusing on fine-grained species classification or delimitation, limited genetic data exist. The finite amount of readily available fine-grained data and the challenges researchers face in material collection, e.g., narrow endemics or remote areas, constitute a significant issue when aiming to finetune state-of-the-art genome foundation models or train large models from scratch. Up-sampling genetic data through DNA augmentation methods has been shown to improve model performance. Typically, these augmentation methods introduce random transmutations, potentially blurring species boundaries and population structures inherent to a given dataset. We examined the potential of an offline augmentation technique that entails interpolating between samples of the same species to add semi-artificial DNA sequences to the dataset. Our approach is transparent, interpretable, and independent from encoding methods or subsequent analyses.

## Introduction

Genetic data have become a fundamental resource for evolutionary and taxonomic research, providing detailed insights into biological diversity across the Tree of Life [1–3]. Throughout the years, technical and methodological advances in sequencing technologies have enabled increasingly large and diverse genetic datasets, ranging from single- to multi-locus to whole genome (re)sequencing [4–6]. In parallel, machine learning (ML) approaches are being applied more and more frequently to these molecular data. These developments, in turn, create opportunities for pattern recognition, classification, and inference in evolutionary biology, systematics, and ecology [7–11].

Genome foundation models such as DNABERT-2 [12], AgroNT [13], GROVER [14], Caduceus [15], Nucleotide Transformer [16], AlphaGenome [17], and Evo 2 [18] are trained on vast amounts of data and have been shown to reliably predict epigenetic marks, splice sites, RNA stability, or gene expression. In works that build genome foundation models, the focus tends to be on human or bacterial DNA and seldom on plant genetics. Oftentimes, models are exclusively trained or even evaluated using non-plant data [12,16,18–20]. When these models are applied to classify species, the prevalent challenges surrounding plant species identification and delimitation are rarely discussed or examined [20]. This includes the high rate of ancient and recent hybridization or allopolyploidy – partly combined with asexuality – that results in intricate reticulate evolutionary patterns in many plant lineages [21–23].

Addressing plant species evolution and relationships requires at least finetuning of existing genome foundation models. However, only coarse-grained genomic data are typically available in databases such as GenBank, where a small number of genomes usually represent entire plant families [6,24]. Subgenomic data such as hundreds of single-copy nuclear genes obtained through target enrichment are more readily available for most flowering plant genera (Kew Tree of Life Project, [25]), but fine-grained subgenomic data with many replicates per taxon for species delimitation and taxonomic purposes are still very limited for many plant groups [26–30]. This is especially true for narrow endemics and remote areas in the high-altitudes or (sub)tropics, for which researchers face very limited sampling sizes. In taxonomic studies, insufficient sample sizes often constitute a significant challenge to the finetuning of pre-trained networks, particularly Large Language Models (LLMs). Augmentation - although not a substitute for sufficient original data - can mitigate suboptimal sample sizes by artificially increasing variation in training data. Primarily popularized in computer vision, data augmentation has since been demonstrated to enhance model generalization across a variety of deep learning domains, including natural language processing [31,32].

Usually, augmentations are applied dynamically during training, which is referred to as online or on-the-fly augmenting [33]. In practice, techniques that use on-the-fly augmenting can be harder to inspect, restricting interpretability, and may limit a researcher’s fine-grained control over how individual samples are transformed [33]. This applies in particular when non-deterministic strategies, such as stochastic or learned augmentations, are used. As a result, samples created through on-the-fly augmentation are usually curated - if at all - post-training. If undesirable effects (e.g., shifts in the empirical data distribution) are discovered only after training has been completed, further finetuning or retraining can be necessary, leading to additional computational cost and time. On-the-fly augmenting may also not be feasible in cases where the target value depends on the augmented input, e.g., when training on genetic distances between pairs of DNA sequences, making augmenting before training (i.e., offline augmentation, [33,34]) the more sensible choice.

For DNA data, augmentations can refer to random mutations, the introduction of insertions and deletions, reverse complementing sequences, translocations, and input value alterations (noise) [35–40]. Even random drawing of sequences from different species that share a common ancestor (homolog), can be considered a form of augmentation [41]. Although approaches injecting indels, mutations, or translocations may model sequences under evolutionary constraints, empirical variation within biological datasets is not necessarily conserved. For instance, probabilistic approaches, such as Hidden Markov Models (HMMs), model sequence variation through learned state transitions and can generate novel sequences by sampling from the resulting distributions [42]. While HMMs can capture complex dependencies, they inherently introduce extrapolation beyond the observed data, as they assign non-zero probability to unobserved sequence configurations. Alternatively, algorithms such as SMOTE [43,44] interpolate on numeric representations of the input. Consequently, interpolated feature values may not map cleanly back to discrete nucleotide states, limiting interpretability and requiring a decoding or reconstruction step. Depending on the research question, neighbor-guided approaches on raw DNA that reflect empirical variation of the underlying dataset may be favored for interpretability or to preserve dataset-specific structures such as distances between or natural variation within species.

To conserve species-defining natural structures and provide an elevated level of transparency, we propose DNAInterpolator, a species-aware and consensus-assisted augmentation method that leverages single nucleotide polymorphism (SNP) variants empirically observed within a dataset. DNAInterpolator augments DNA by interpolating between DNA samples belonging to the same species and exploits consensus sequences to limit genetic divergence in newly generated samples. Interpolating between samples ensures that only naturally occurring mutations, insertions, and deletions are created during sequence simulation. Consequently, our approach does not aim to model sequence evolution. Instead, it represents a conservative, strictly data-constrained interpolation strategy that allows for back-tracing of injected information within generated samples while limiting deviations based on empirical variation. By preserving intrinsic biologically distinguishing information, employing context-aware augmentations may benefit plant species identification and delimitation efforts.

Plant species delimitation, in particular, may also be improved by a biologically meaningful arrangement of learned representations (embeddings) in the model’s latent space. Biologically meaningful embeddings are characterized by a spacing of sequence embeddings that mirrors the biological (e.g., genetic) distance and can be used to infer phylogenetic relationships. To achieve such a distribution of embeddings within the embedding space, a model can be explicitly trained on genetic distances [7]. For the prediction of phylogenetic distances from unaligned data, multiple embedding-free methods have been proposed [45,46]. In contrast to these approaches, ML focuses on representation learning, which aims to map sequences into a continuous embedding space whilst decreasing the dimensionality significantly. Since representations are learned, they are inherently applicable to a broad range of tasks, such as taxonomic assignment [7], functional analyses [36], and species delimitation [23]. While alignment-free methods provide direct distance estimates for a given dataset, they do not yield a transferable representation.

Outside the field of ML, genetic distances remain fundamental to species identification and delimitation efforts [47]. They allow for quantification of relatedness between samples, serve to infer phylogenetic structure, and help delineate biological boundaries [23]. Given their established role in taxonomic research, incorporating genetic distances into ML frameworks is a natural extension and has already been applied in various studies [7,48,49]. However, unequal distribution of targets, e.g., class imbalance, is known to raise issues in the training of ML models [50,51]. Since the distribution of genetic distances within training datasets may be heavily biased towards almost identical or highly divergent samples, we suggest using the interpolation of sequences and the accompanying increase in dataset size to level the distribution of genetic distances within the training dataset.

In this study, we examined whether training with an dataset interpolated using DNAInterpolator improves the prediction of genetic distances (as a proxy to biologically meaningful embeddings) compared to training using a non-interpolated dataset. We then tested whether balancing interpolated data improves distance predictions compared to non-interpolated and interpolated data. To this end, we trained on three augmentation approaches separately: a) non-interpolated, b) interpolated, and c) interpolated and balanced. To illustrate the general applicability of our approach, we evaluated all interpolation and balancing schemes on four recently revised plant genera spanning orchids, herbaceous lineages, and tropical trees.

## Materials and methods

### Datasets

**Fig 1:**
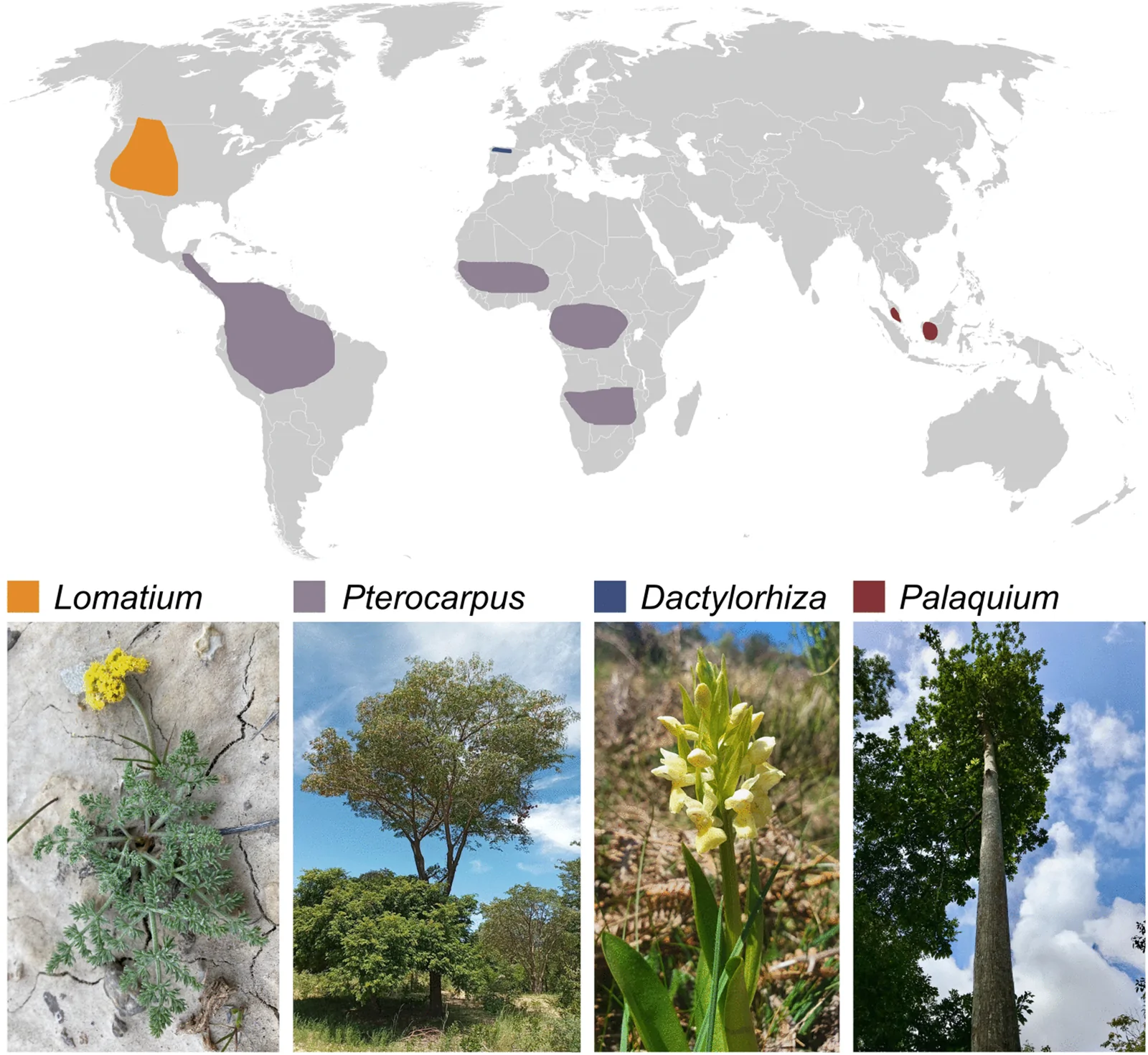
Global distribution of dataset samples. Origins of samples belonging to the four plant genera datasets *Lomatium* [29] (https://www.inaturalist.org/observations/214305391 by Cecelia Alexander, 2024; no rights reserved), *Pterocarpus* [28] (https://www.inaturalist.org/observations/149125232 by dune_ninja, 2023; no rights reserved), *Dactylorhiza* [30] (https://www.inaturalist.org/observations/115884731 by Martiño Cabana Otero, 2022; CC-BY 4.0), and *Palaquium* [27] (https://www.inaturalist.org/observations/37642912 by Nasser Halaweh, 2020; CC-BY 4.0). Public domain map by Canuckguy et al., obtained from Wikimedia Commons (https://commons.wikimedia.org/wiki/File:BlankMap-World-Compact.svg).

In this study, we applied all interpolation and balancing schemes on phylogenomic datasets generated with the Angiosperms353 probe set, a widely used nuclear gene sequencing framework designed for universal application across flowering plants [52]. The Angiosperms353 probe set provided a standardized basis for training and allowed for testing on the same loci used for training. At the time of conducting the experiments, few comprehensive studies existed that used the Angiosperms353 probe set and provided a reasonable amount of data (i.e., yielding data on multiple individuals per species) for interpolation and training. Here, we trained and evaluated on four datasets, each representing samples from distinct plant groups. Information on the number of individuals and loci within each dataset can be found in Table 1, while information on the geographic distribution of the sampling locations for the four groups is shown in Fig 1.

**Table 1:** Training and validation subset sizes of plant group datasets. List of datasets used for training and validation. Provided are the number of species, original individuals within the dataset (total, with outgroups in parentheses), training (train) and validation (val) subsets, loci present within the respective dataset, the mean number of SNPs per locus, and the average pairwise p-distance observed within the respective dataset. σ = standard deviation.

Genus	Species	Individuals/ Species (σ)	Individuals			Loci	SNPs/ Locus	Mean pairwise p-distance
total	train	val
***Dactylorhiza***	3	6.67 (0.58)	20	13	7	266	68.02	0.29
***Lomatium***	5	3.88 (4.63)	51(+11)	40	22	286	592.95	0.25
***Palaquium***	8	10.5 (4.72)	95(+9)	68	36	260	114.1	0.01
***Pterocarpus***	12	11.07 (9)	153(+1)	104	50	319	533.86	0.06

The four plant groups include *Dactylorhiza*, which belongs to the Orchidaceae family [30]. Orchids have long-since been established as model organisms for the study of intricate evolutionary processes. Alongside substantial morphological and ecological diversity, the family exhibits reticulate evolutionary patterns. The genus *Dactylorhiza* exemplifies this complexity: extensive hybridization and recurrent formation of allopolyploid lineages are followed by diversification through ecological adaptation [53]. As a result, species boundaries and phylogenetic relationships within *Dactylorhiza* are often difficult to disentangle. The *Dactylorhiza* dataset focuses on *D. cantabrica*, a recent hybrid species endemic to the northwest of the Iberian Peninsula. Also included in the dataset are its parents *D. sambucina*, a Eurosiberian-Submediterranean diploid species, and *D. insularis*, a triploid species native to southern Europe. All three species considered in this study belong to the Sambucinae section, which is morphologically well characterized, although its constituent species are not. In total, the dataset covers 20 individuals across the three species with 266 loci sequenced using the Angiosperms353 nuclear gene probe set.

The second dataset examined in this study comprises samples from the genus *Lomatium* [29], a member of the perennial endemic North American clade of Apiaceae (PENA). PENA species are herbaceous perennials and most often occur in the western United States. They display a high degree of morphological uniformity across taxa. This similarity, combined with narrow endemism, sympatry, variable diagnostic traits, and frequent misidentifications, has long complicated species delimitation and comparative studies within the group. Within PENA, the *Lomatium foeniculaceum* species complex represents the most widespread lineage, extending across much of western and central North America. Its morphology appears as a continuum, impeding efforts to delineate species. Based on 286 Angiosperms353 genes, 5 species with one species comprising two varieties have been described for the *L. foeniculaceum* complex and are included in the dataset.

*Palaquium*, the third dataset, is a member of the Sapotaceae family, and a genus of tropical tree species that can be found anywhere from India to Australia, and the western Pacific [27]. Its diversity hotspot lies in Malesia, where all samples from this dataset were collected. Sapotaceae species are usually diploid, with few polyploid members mostly occurring within the tribe *Sideroxyleae*. The *Palaquium* dataset therefore includes data from mostly diploid individuals (except for *P. obovatum* found to be hexaploid in [54]), comprises 8 species in total and covers 260 loci from the Angiosperms353 probe set.

The fourth and last dataset comprises samples from *Pterocarpus*, a genus of the Fabaceae family [28]. *Pterocarpus* representatives are trees with a pantropical geographic distribution. Within *Pterocarpus*, species delimitation is complicated by high morphological variability and intermediate characteristics. The dataset contains data from 12 species native to Africa, with two of them also occurring in the Neotropics. The genetic data comprises 319 Angiosperms353 genes.

### Interpolation

In this study, interpolation is characterized by the transfer of SNPs from one genetic sample (donor) to another (recipient). More specifically, we implemented a species-aware consensus-based (SACB) interpolation technique using Python v3.13.2. An overview of the SACB interpolation workflow is given in Fig 3 with pseudo-code shown in Alg. 1. Detailed information about how to run the pipeline and what customization options are available to the user can be found in the code’s README and on the GitHub page.

**Fig 2:**
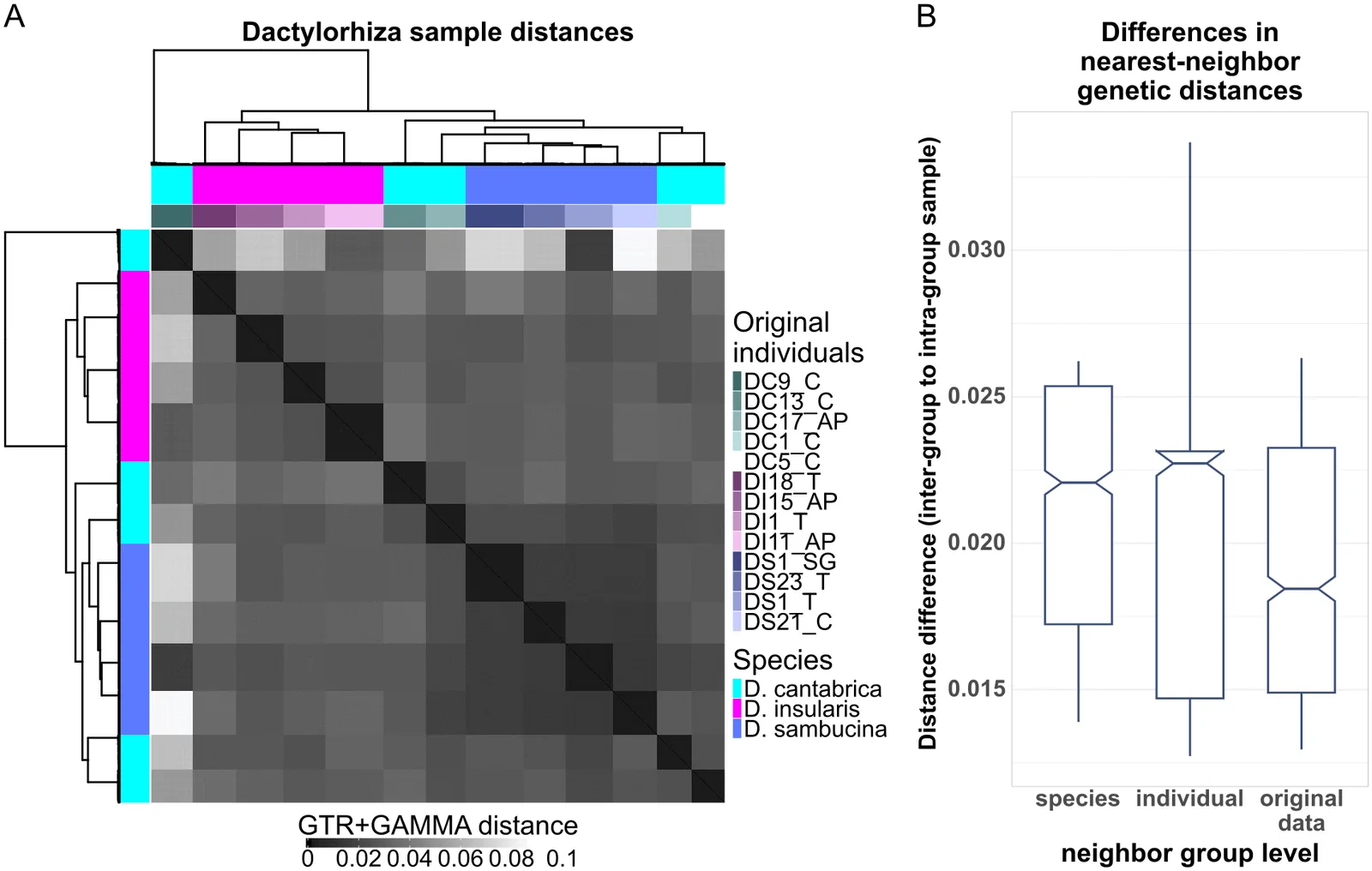
Clustering of GTR+GAMMA distances of interpolated and original *Dactylorhiza* samples. A) Shown are GTR+GAMMA distances calculated with RAxML between original and interpolated samples. Each sample is a concatenated sequence of loci that represents an individual. Missing loci were filled with gaps. Colored bars at the top and to the left of the heatmap illustrate species information. The thinner colored bars on top of the heatmap directly below the species information represent original individuals. Interpolated individuals are colored according to the respective original individual (recipient) used during interpolation. B) Shown are differences between genetic distances of nearest neighbors across and within groups. For instance, data points for ‘species’ include differences between genetic distances of a) individuals belong to the same species, and b) individuals belong to different species. For original data, only genetic distances from interpolated samples to a) the respective original sample, and b) unrelated original samples were considered. Both plots are based on a subset of 1000 original and interpolated samples, where interpolated samples were randomly chosen.

**Fig 3:**
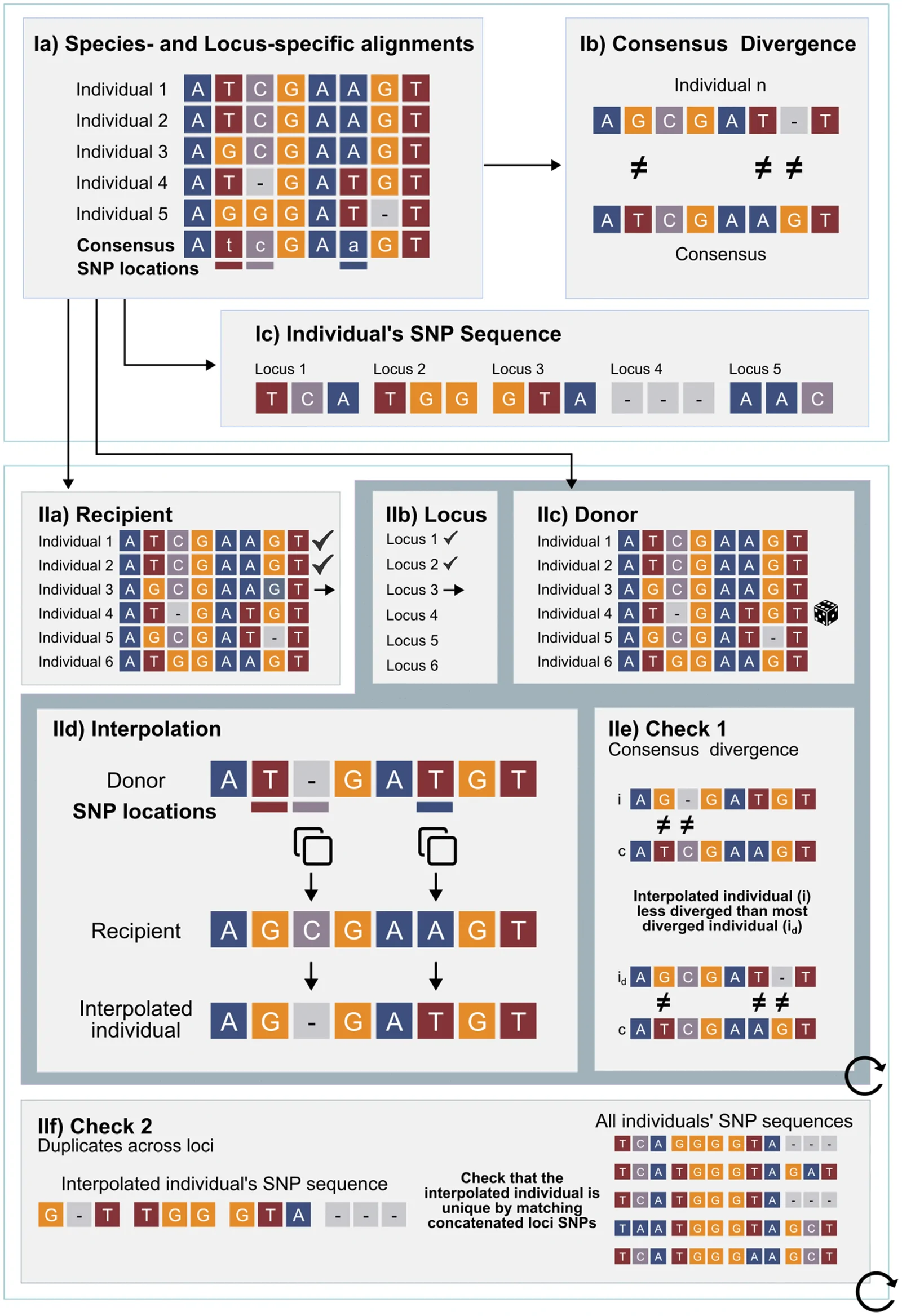
Workflow of the interpolation pipeline. Illustrated are the steps included in the interpolation pipeline. The pipeline starts with species- and locus-specific alignments from which consensus sequences are calculated (Ia). Per individual, the divergence of each DNA sequence to the species- and locus-specific consensus as well as the SNPs across all loci are saved (Ib and Ic). We interpolate by iterating over all individuals within the dataset as well as all loci (IIa and IIb). Per iteration, a donor sequence is determined at random (IIc). We choose a subset of SNP positions to copy bases from the donor to the recipient sequence, creating a new, interpolated DNA sequence (IId). We then check that the new sequence does not differ from the consensus sequence at more positions than the most diverged sequence (IIe). At last, we check for duplicates by comparing the SNP sequence of the newly generated individual with the SNP sequences of all other individuals within the dataset (IIf).

The SACB interpolation pipeline starts with creating FASTA files for each species-locus group. To avoid generating augmented sequences that incorporate site-specific variation from the validation data or create intermediate sequences bridging training and validation samples, we determine the splits before augmentation and interpolate using only the training subset. Based on the generated FASTA files, we calculate p-distances using the R package ape (versions 4.4.2 and 5.8, respectively) and store them for later retrieval of unmodulated position-based differences between sequence pairs [55]. The group-specific FASTA files also serve as a basis for generating consensus sequences, both for the individual loci and for the loci-concatenated individuals.

For each individual, we build a SNP sequence by iterating over all loci present within the dataset and accumulating the SNPs per locus. SNPs are determined based on the generated FASTA files using snp-sites version 2.5.1 [56]. If a locus does not contain any SNPs, the locus is skipped. If there is no sequence for a given locus and a given individual, the position within the SNP sequence of that individual is padded with as many gaps as there would have been SNPs. The SNPs sequences are stored alongside the p-distances for later usage.

After preparing the p-distance and SNP data, the interpolation begins. Our interpolation approach aims to generate N new individuals per original individual, where N refers to a user-defined number. The number of individuals to generate depends on the desired dataset size and how strict downstream filtering is applied. Depending on the number of SNPs and variants, there will also be a threshold to how many sequences can be generated. To prevent infinite loops if the pipeline is unable to meet the requested number of interpolated individuals due to failure of downstream sequence checks (see Fig 3), we added patience, breaking the iteration and continuing with the next individual if 10 consecutive tries at interpolating are unsuccessful. The patience can be adjusted by the user. A reasonable value for patience within DNAInterpolator is closely linked to how similar the original input DNA sequences are and can be lowered to ensure a shorter runtime or increased to accommodate for almost identical DNA sequences. In general, the chosen settings may impact the success of downstream ML training through the size of the training dataset.

When trying to generate new individuals, we shuffle the dataframe containing all records to prepare for later random sampling of an interpolation partner. The shuffled samples are then grouped by locus and iterated over. Grouping by locus allows us to create a new sequence for each locus while having all potential interpolation partners at hand.

When choosing an interpolation partner, we ascertain that the chosen sequence differs from the original sequence by restricting the shuffled record dataframe to sequences with a p-distance of non-zero. We then randomly sample an interpolation partner. The interpolation partner acts as a donor during interpolation. The recipient and donor sequences are reduced to the SNPs that differ between them. With SACB, we further reduce the relevant SNPs to the recipient positions that already diverge from the consensus whenever additional deviating positions would exceed the maximum divergence from the consensus sequence observed within the dataset. This reduction ensures that the generated sequence remains consistent with the genetic distance to the consensus empirically observed within the dataset, thereby preventing a potential expansion of the sequence space.

To finalize the number of bases to alter, we use a skewed random distribution. The minimum number of alterations is 1 while the maximum number is either the maximal deviation from the consensus or the number of alterations left for the complete individual when taking into account the already altered bases of other loci. To not exceed the divergence from the complete-individual consensus, the distribution is skewed towards fewer alterations, preserving sufficient deviations for the loci yet to be processed.

Based on the determined number of alterations, we sample SNP positions from the filtered list of SNPs. The sampled SNP positions are then copied from the donor sequence to the recipient sequence. We store the SNPs across all loci in a SNP sequence that is later used to check for duplicate individuals. If there is no sequence for a specific locus for the current individual, we again create a gap-only padding for the SNP sequence. Depending on the dataset and the desired number of individuals to be generated, interpolation per dataset used in this study took up to 5 hours on an M1 MacBook Pro with 16 GB of RAM and 4 CPU cores. Formatting of interpolated data for training took an additional maximum of 11 hours using 160 GB and 4 CPUs.

To examine whether interpolated and original individuals from the same species clustered together, we sorted and concatenated all loci belonging to the same individual. Missing loci were filled with gaps. We then ran hclust of the base R stats package with ‘complete’ as the clustering method. The output was given to the function Heatmap, included in the ComplexHeatmap package v.20.2 [57]. The heatmap representations of generated interpolated sequences per plant group can be found in Fig 2A, and in panel A of A-C Figs in S1 Appendix. In addition, we visualized the difference between the nearest neighbor of a sample within and across different levels of groups (Fig 2B, and panel B of A-C Figs in S1 Appendix). The groups cover samples belonging to the same or a different species, the same/different recipient individual, and samples’ genetic distances to the corresponding original recipient individual and the most closely related non-corresponding original recipient individual. The differences were calculated by subtracting the distance to the nearest in-group sample from the distance to the nearest out-of-group sample, i.e., positive values indicate a closer relationship to in-group samples.

**Algorithm 1. Sequence interpolation**

**Require:** Input sequences *S*, species labels $L_{sp}$, individual labels $L_{i}$, locus labels $L_{l}$

1:  **for** species *s* in $L_{sp}$ **do**

2:   **for** locus *l* in $L_{l}$ **do**

3:    Get subset of sequences *S* belonging to species and locus $\left(S_{sl}\right)$

4:    **for** sequence pair $\left(s_{i},s_{j}\right)$ in $S_{sl}$ **do**

5:     Compute p-distances $d_{sl\_ij}$

6:    Determine SNP positions $s_{sl}$ based on $S_{sl}$

7:    Generate consensus sequence $c_{sl}$ based on $S_{sl}$

8:    Determine maximum divergence from consensus $c_{sl}$ within $S_{sl}$ $\left(cd_{s}l\right)$

9:   **for** individual *i* in $L_{i}$ **do**

10:    **for** locus *l* in $L_{l}$ **do**

11:     Get locus sequence *s* belonging to individual

12:     Determine divergence of *s* from consensus $c_{sl}$

13:    Add divergences to determine across-loci divergence from consensus

14:   Determine maximum divergence across loci $cd_{s}$

15:  **for** individual (*i*) in $L_{i}$ **do**

16:   Get subset of *S* belonging to *i* $\left(S_{i}\right)$

17:   Get species *s*

18:   **for** number of individuals to interpolate (*n*) **do**

19:    Initialize number of across-loci changes $nc_{a}=0$

20:    Initialize SNPs of interpolated sequences across loci $snps_{i}$

21:    **for** recipient sequence $\left(s_{r}\right)$ in $S_{i}$ **do**

22:     Get locus *l*

23:     Determine possible donors $\left(i_{ds}\right)$ belonging to *s*

24:     Determine donor individual $\left(i_{d}\right)$ from $i_{ds}$ where $d_{sls_{r}s_{d}}>0$ and $i_{d}!=i$

25:     Get donor sequence $\left(s_{d}\right)$ based on $\left(i_{d}\right)$ and *l*

26:     **procedure** Determine maximum number of positions to change

27:      Get maximum divergence from locus consensus $cd_{sl}$

28:      Get maximum divergence across loci $cd_{s}$

29:      Initialize maximum positions to change as $min(cd_{sl},cd_{s}-nc_{a})=p_{max}$

30:     Add number of changed nucleotides to $nc_{a}$

31:     Get list of SNP positions (*snps*)

32:     Get list of SNP positions where value diverges from consensus $\left(snps_{d}\right)$

33:     Remove identical positions between $s_{d}$ and $s_{r}$ from *snps*

34:     Determine divergence of $s_{r}$ from consensus $c_{sl}$ $\left(cd_{r}\right)$

35:     **if** $cd_{sl}==cd_{r}$ **then**

36:      Reduce SNP positions of *snps* to positions where $s_{r}!=c_{sl}$

37:     Determine number of positions to change $\left(p_{nc}\right)$ within a range of $1,p_{max}$ by considering a random distribution

38:     Randomly sample $p_{nc}$ positions from *snps* to change $\left(p_{c}\right)$

39:     Copy $p_{c}$ from $s_{d}$ to $s_{r}$ to generate interpolated sequence $\left(s_{i}\right)$

40:     Save SNPs of $s_{i}$ as $snps_{il}$

41:    Concatenate SNPs $snps_{i}l$ to create $snps_{i}$ and match against original and generated individuals $\left(S^{\prime }\right)$

42:    **if** $snps_{i}$ is in $S^{\prime }$ **then**

43:     Discard interpolated sequences $s_{i}$ of *n*

44:     **if** interpolation was unsuccessful for the last 10 times **then**

45:      break and process next individual

46:    **else**

47:     Store $snps_{i}$

### Assembling of training data

#### No interpolation.

To build the dataset without any interpolation, we read the original per-locus FASTA files. From the FASTA files, we stored the genetic sequence, the individual’s ID within the sequence header, and the locus within a dataframe. We calculated ground truth genetic distances using RAxML v8.2.12 with GTR+GAMMA as its substitution model, thereby incorporating evolutionary information beyond simple site-wise differences [58]. The sample dataframe was then merged with the genetic distances, resulting in a dataframe that encompasses a pair of sequences belonging to the same locus in each row. Duplicate sequence pairs were removed. We further filtered the sequences to be at least 50% of the median sequence length. Sequence pairs should also have at least a 95% overlap, i.e., we should encounter a non-gap in both sequences at 95% of positions along the aligned sequence length.

We converted raw genetic distances to cosine distances for a bounded and stable training signal. The transformation from genetic distance to cosine distance is monotonic and invertible, meaning that, in principal, approximate GTR+GAMMA distances can be recovered from the learned sequence embeddings. However, the objective of our approach is not the precise reconstruction of genetic distances, but rather the preservation of their relative relationships within the embedding space. Consequently, cosine distances should not be interpreted as direct estimates of genetic distance.

Our methodology closely follows existing similarity learning approaches (e.g., [20]), where pairs of similar sequences (positive pairs) are drawn together and dissimilar sequence (negative) pairs are pushed apart during training. We modified these approaches with soft labels representing genetic distances. While cosine similarity is widely used in similarity learning [20], cosine distance allows genetic distances to be modeled directly as distances in the learned embedding space, rather than indirectly via similarity scores. Cosine similarity ranges from -1 (maximum dissimilarity) to 1 (identical) while cosine distance ranges from 0 (identical) to 2 (maximum distance). To convert the genetic distances, we first determined the distance that accounts for 99.9% of all entries across plant groups. This distance was then set to equal 2, with proportionate rescaling of all distances according to this new maximum. Sequence pairs with a cosine distance larger than 2 were removed from the dataframe to mitigate potential downstream effects of very large outlier distances and prevent sparsely populated sub-ranges of the distance distribution. Since the cutoff is percentile-based, it scales with the distances comprised within the respective dataset. Nevertheless, we recommend examining the pairwise-distance distribution as well as the distance range relevant to the intended inference data to ensure sufficient overlap in distributions. Where the inference data cover a substantially narrower distance range than the training set, adjustment of the cutoff is recommended.

**Fig 4:**
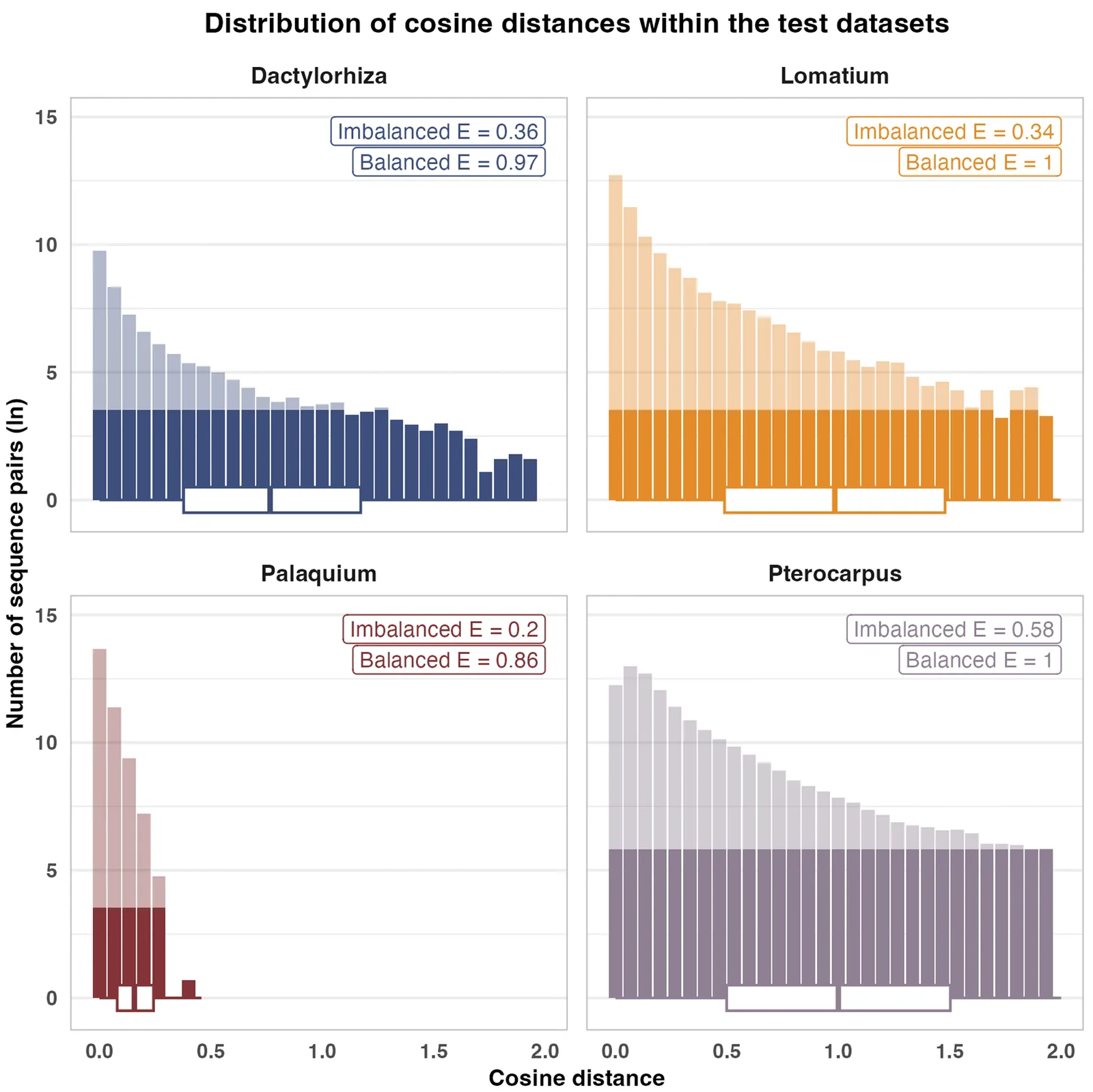
Distribution of distances within the test datasets. Shown are the log-transformed number of test samples per dataset. Dark colors represent a balanced subset of test samples, with an approximation to a uniform distribution of distances. Boxplots indicate the distance distribution of the balanced test sets. Included are normalized entropy values (E) for both the complete test set and the balanced subset. Higher entropy values indicate a more uniform distribution.

To partition the training and validation subsets, we looped over a shuffled list of individuals and sequentially added them to the validation subset. The added individuals were then not used within the training, i.e., individuals within the training and validation subsets were mutually exclusive. For each iteration, we calculated the proportion of validation samples and stopped when the percentage exceeds 20%. Since subsets were based on combinations of sequences and the number of loci per individual varies, the number of sequence pairs that was transferred to the validation subset or erased from the combined dataset varies. Hence, the ratio of training to the validation subset varies between plant group datasets. The portion of the validation samples compared to the entire dataset ranges from 20.25% for the *Pterocarpus* dataset to 25.11% for the *Dactylorhiza* dataset. An overview of the number of samples within the datasets and subsets across interpolation approaches is given in S1 Table. To ensure a balanced evaluation across the distance range, we subset the training and applied normalized entropy as a metric for uniformity using the entropy package v1.3.2 [59]. The number of test samples is detailed in Fig 4.

#### Imbalanced interpolation.

Creating the dataset with imbalanced interpolation followed the same steps detailed in Section No interpolation. These steps entailed the reading of sequences from the FASTA files that contain the original as well as the interpolated sequences. The sequences were filtered to be at least 50% of the median sequence length and the sequence pairs must overlap at a minimum of 95% of the aligned positions. After filtering, we selected an equal number of sequence pairs from all genera, matching the maximum number observed among the non-interpolated datasets. In this study, the *Pterocarpus* training subset contained the highest number of non-interpolated sequence pairs (630,010). Accordingly, we sampled 630,010 sequence pairs from each genus-specific dataset. Sampling was performed in an augmentation-aware manner, where original sequences were chosen first. The gap to the requested number of sequence pairs was then closed by random sampling of pairs that include interpolated sequences. The training on interpolated but imbalanced data used the same validation samples as the training on non-interpolated data.

#### Balanced interpolation.

**Fig 5:**
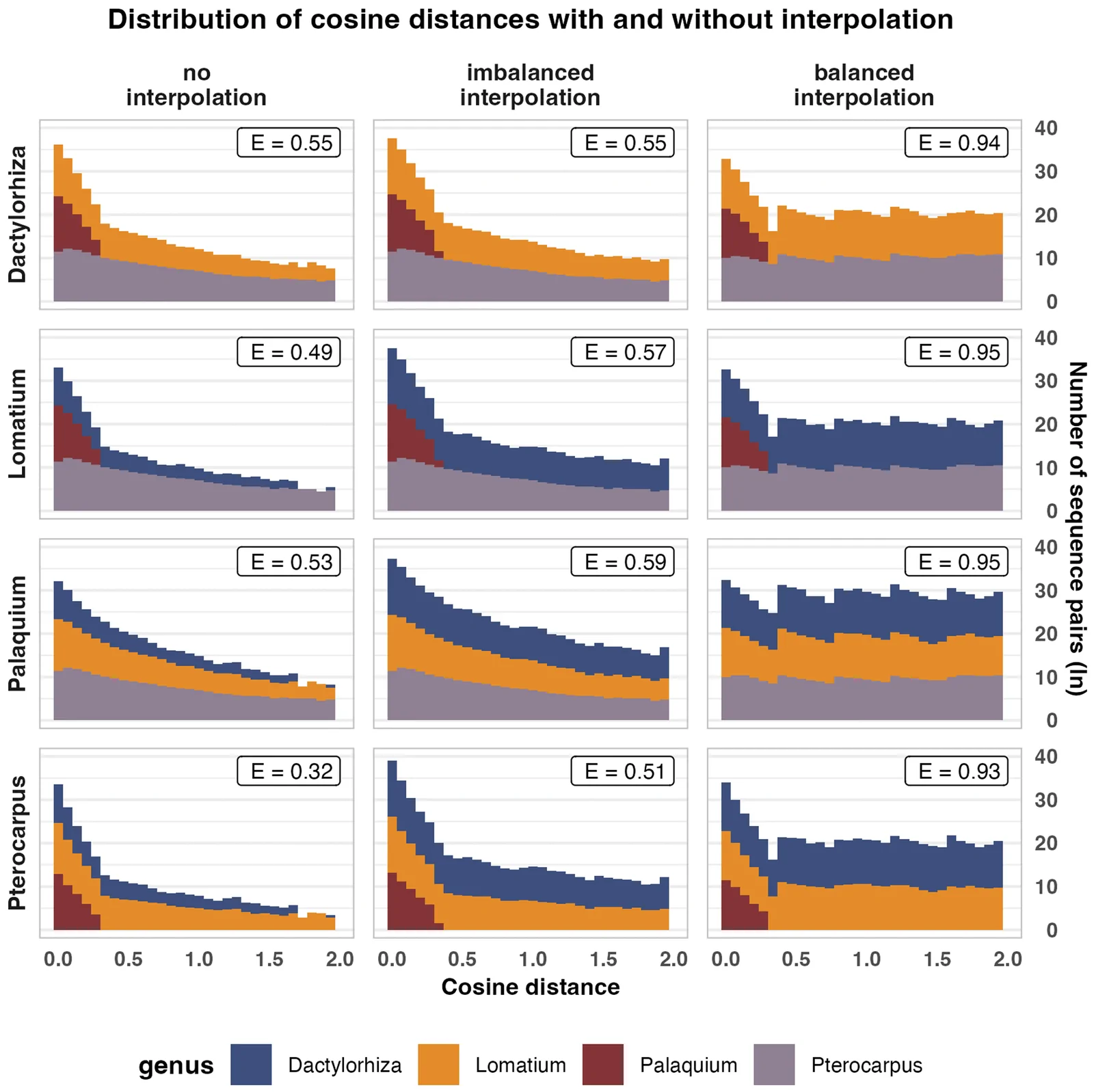
Distribution of cosine distances per LOOCV with and without interpolation. Shown are the log-transformed numbers of sequence pairs per Leave-One-Out Cross-Validation (LOOCV) run and interpolation method. Colored segments within the bars indicate the number of samples belonging to the corresponding genus within the training dataset. For instance, only data from *Lomatium*, *Palaquium* and *Pterocarpus* are used in training during the *Dactylorhiza* LOOCV run. While the number of samples within the datasets using imbalanced interpolation was increased without controlling the distance distribution, balanced interpolation aimed for a mostly equal distribution of genetic distances. Included are normalized entropy values (E) across LOOCV training sets. Higher entropy values indicate a more uniform distribution.

Creating training sets with interpolated and balanced data leveraged the already prepared unfiltered and not yet downsampled records collected during the generation of the interpolated but imbalanced data described in Section Imbalanced interpolation. To balance out the distance distribution within the interpolated training subsets, we assigned a sector to each sequence pair based on the corresponding genetic distance. In total, we opted for five fixed-width sectors across the cosine distance range, striking a pragmatic balance between granularity and the number of samples that fall within the defined thresholds. While the number of bins can influence the distance distribution on a fine-grained level, our goal was to assess whether a more balanced coverage of the genetic distance range improves generalization. This higher-level effect is not expected to depend critically on the precise number of bins. For each bin and training run, the pipeline randomly sampled sequence pairs from the complete dataset. The algorithm selected an equal number of records per bin and maintained the same total number of records across all training runs, including the interpolated distribution-balanced and the interpolated but distribution-imbalanced runs. The change in the distribution of genetic distances between interpolation methods is shown in Fig 5. Again, the validation was performed on the same samples as when training on non-interpolated records.

### Training

To test the effect of our interpolation regime and the balancing of genetic distances, we implemented a Leave-One-Out Cross-Validation (LOOCV). In this instance, LOOCV refers to training on three of the four datasets, validating on left-out data of the same datasets, and testing on a fourth dataset that was neither trained nor validated on. For instance, we trained one model on sequence pairs belonging to *Dactylorhiza*, *Lomatium*, and *Palaquium* while validating on specifically assigned validation samples from those same datasets. The trained model was then tested on data from *Pterocarpus*. While we could have opted for training using a classic training/validation/test split, assigning a subset of samples to an independent test set would have subtracted the corresponding individuals from the set used for interpolation and training to prevent information leakage. Consequently, a separate test split would have substantially reduced the number of sequence pairs within the training set. LOOCV at a dataset level allowed us to maximize the number of sequence pairs per plant group used for training, while also providing the model with the additional challenge of predicting distances between sequences of unseen genera. All our trainings used BERT as the network architecture and pre-trained weights from the genome foundation model DNABERT-2 [12,60]. In addition, we tokenized our DNA sequences using the DNABERT-2 tokenizer. Since the DNABERT-2 tokenizer was built using genomic DNA and is unable to translate gaps, our models did not encounter gaps within the input.

Our general training script followed MosaicBERT as this is also the basis for DNABERT-2 [61]. We adjusted the model and loss function to accommodate our pairwise sequence input. After forwarding the two tokenized DNA sequences through the network, we determined the cosine distance of the resulting embeddings. The cosine distance was then compared with the target cosine distance which was converted from the raw genetic distance, yielding the training loss. We used a global training batch of 256 with a learning rate of 1.6e-5, which we linearly adjusted from the learning rate of DNABERT-S, a species-aware model based on DNABERT-2 that leverages similarity learning and is therefore closely related to our paired sequence approach [20]. Each LOOCV training took approximately 3 days running on 6 NVIDIA A40 GPUs.

### Evaluation

We tested the trained models using all combinations of samples from the genus left out in the training. The number of evaluated DNA sequence pairs, along with information on how many combinations comprised sequences of the same (intraspecific) or different (interspecific) species, can be found in Table 2.

**Table 2:** Number of evaluated DNA sequence pairs per dataset. List of datasets used for evaluation. Provided are the number of DNA sequence pairs with shared and with different species.

Genus	Total	Intraspecific pairs	Interspecific pairs
***Dactylorhiza***	794	234	560
***Lomatium***	1004	139	865
***Palaquium***	173	16	157
***Pterocarpus***	10200	828	9372

To determine how well the model predicts genetic distances between DNA sequences, we first normalized the predicted cosine distance and the ground truth cosine distance transformed from the raw genetic distance. The normalized predicted and target distances were then subtracted and the absolute value was determined. For statistical comparisons of distance predictions between interpolation methods, we used R v4.4.2 [62] and applied the non-parametric Friedman test for paired data. We used a paired Wilcoxon test for the post-hoc analysis. For the analysis of the influence of the interpolation method on the divergence of genetic distance predictions to the ground truths across the entire range of the cosine distance, we employed a Linear Mixed-Effects Model using the lmerTest package v3.1 in R, in combination with a Type III ANOVA with Satterthwaite’s method to account for unbalanced data. For the post-hoc analysis, we used estimated marginal means (least-squares means) through the emmeans package v1.10.4. For comparisons of correlations between predicted and target distances, we calculated the Spearman’s rank correlation coefficient. Results are plotted using ggplot2 v3.5.2.

## Results

### Distance distribution after interpolation and balancing

We trained models using original data, interpolated data, and interpolated data with a balanced distribution of genetic distances. Fig 5 visualizes the genetic distance distributions of our three augmentation approaches. The sample size of the datasets with imbalanced interpolation increased in comparison to the datasets without any interpolation (S1 Table). However, the distribution of distances across the cosine distance range remained mostly consistent, with a strong bias towards smaller distances in all four LOOCV training sets. Training without *Dactylorhiza*, median distances were ~0.07 for no interpolation, ~0.05 for imbalanced interpolation, and ~0.97 for balanced interpolation. Leaving out *Lomatium*, the raw dataset had a median distance of ~0.08, the imbalanced interpolated dataset had a median of ~0.06, and the balanced interpolated dataset a median of ~0.97. When excluding *Palaquium*, median distances ranged from ~0.11 without interpolation, across ~0.07 for imbalanced interpolation, to ~0.98 for balanced interpolation. We observed the smallest median genetic distance in the training without *Pterocarpus*, with ~0.02 for no interpolation, ~0.03 for imbalanced interpolation, and ~0.99 for balanced interpolation. The detailed examination of sample contribution per group shows that *Palaquium* exclusively yields sequence pairs with especially small genetic distances, upholding an unequal distance distribution even in datasets with balanced interpolation Fig 5. Still, the datasets with balanced interpolation exhibit a notably more equal distribution compared with both none and imbalanced interpolation.

### Predicted distance divergence

**Fig 6:**
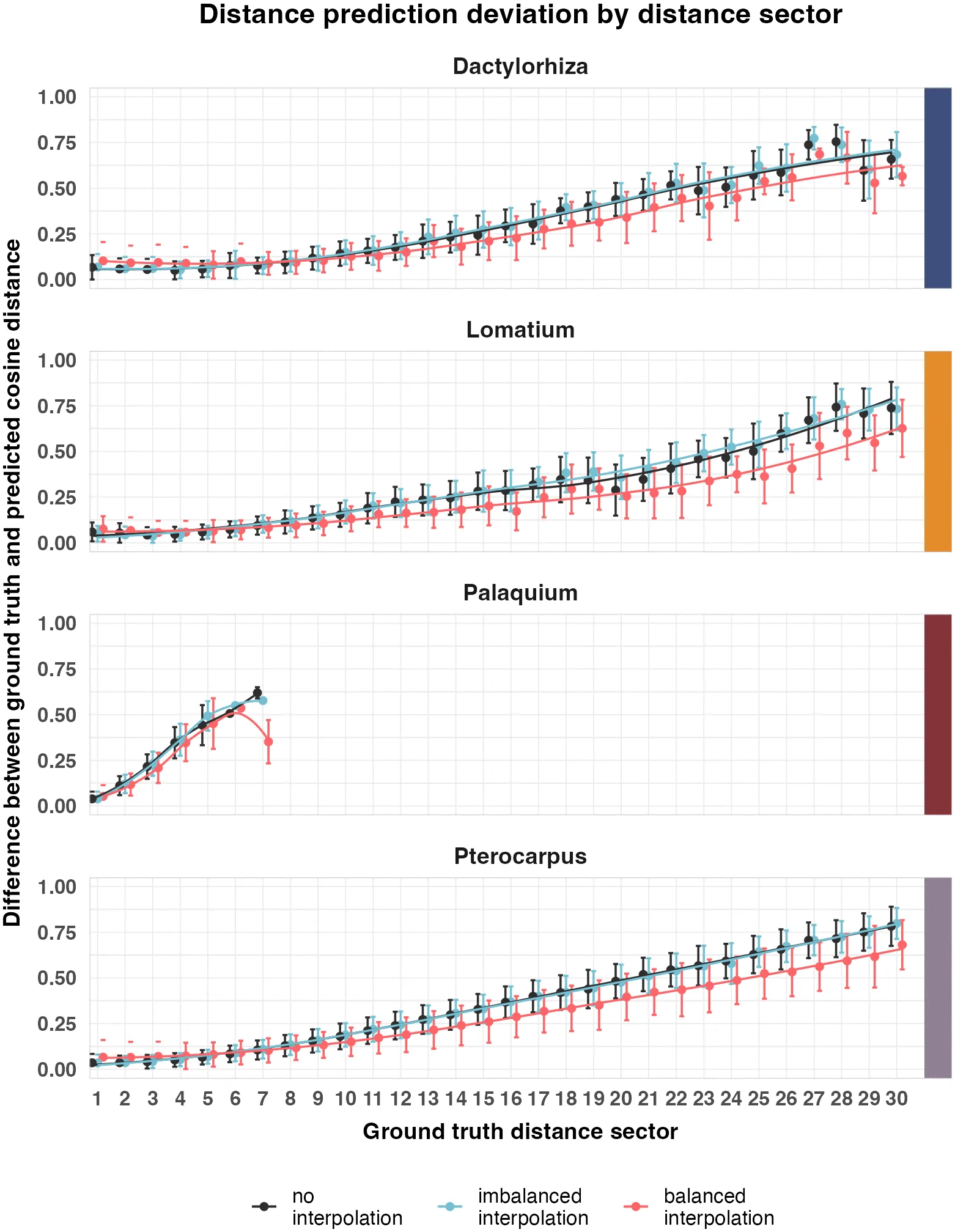
Divergence between predicted and ground truth cosine distances. Illustrated are the absolute differences between predicted and ground truth cosine-transformed normalized genetic distances for 30 linearly placed sectors across the cosine distance range. The sectors are represented by the mean differences between the predicted and ground truth distances. Lower predicted to ground truth differences indicate better model performance. Each facet shows the results of a LOOCV run, with testing on the left-out dataset. The results for the interpolation methods are shown side-by-side and are distinguishable by color.

Our training workflow using LOOCV allowed us to test the three interpolation approaches on all four plant groups within our collective dataset. For our evaluation, we calculated the absolute divergence between the ground truth and the predicted cosine-transformed and normalized genetic distance. The results are illustrated in Fig 6. We observed significant differences in genetic distance prediction divergence from the ground truth between interpolation methods for all four datasets when considering data from all sectors, i.e., the complete test set (Friedman p < 0.001; Friedman chi-squared: *Dactylorhiza* - 66.2, *Lomatium* - 54.3, *Palaquium* - 14.8, *Pterocarpus* - 623.8).

Imbalanced interpolation was outperformed by no interpolation in *Dactylorhiza* (pairwise Wilcoxon p < 0.001). In *Lomatium*, training with imbalanced interpolation yielded improved distance prediction compared to training without interpolation (pairwise Wilcoxon p < 0.001). In *Palaquium* and *Pterocarpus*, imbalanced and no interpolation provided comparable results.

When balancing interpolated data, we found that for *Dactylorhiza* and *Palaquium*, using only original DNA sequences yielded cosine distances between embeddings that most closely resemble ground truth genetic distances (pairwise Wilcoxon p < 0.001). Testing on *Dactylorhiza*, imbalanced and balanced interpolation showed comparable distance deviations while providing slightly less consistent distance predictions compared to the ground truth than the training on non-interpolated data. In *Palaquium*, the training on imbalanced data yielded results in between the non-interpolated and balanced trainings. For *Lomatium* and *Pterocarpus*, the significantly smallest divergences from the target distances were provided by training based on the balanced interpolation approach (pairwise Wilcoxon p < 0.001). In *Lomatium*, non-interpolated data, therefore, yielded the highest differences between predicted and target distances.

Taking into consideration differences in performance across the cosine distance range, we applied an ANOVA on the fixed effects of a linear mixed-effects model and found that the ground truth distance, the interpolation method, and their combination significantly influenced the divergence of the model’s prediction from the ground truth (ANOVA p < 0.0001). In *Dactylorhiza*, a post-hoc estimated marginal means pairwise comparison between interpolation methods showed that, for small distances (sectors 1–7), not using interpolated samples yielded the most accurate distance predictions (p < 0.0001), while balanced interpolation significantly outperformed imbalanced and no interpolation for medium to large target distances (sectors 8–30, p < 0.0001). In *Lomatium*, imbalanced interpolation performs best for small distances (p < 0.0001), while balanced interpolation has the lowest divergence between ground truth and target distance when considering larger target distances (p < 0.0001). As only data within sectors 1–7 are available for *Palaquium*, no test was performed on larger distances. For smaller distances, not applying interpolationg resulted in the most accurate distance predictions (p < 0.0001). *Pterocarpus*, similar to *Dactylorhiza*, performs best not applying interpolation when predicting small distances (p < 0.0001) and balancing interpolation when predicting larger distances (p < 0.0001).

### Predicted distance correlation

For a more detailed look at the performance of the different models across the full range of genetic distances within the datasets, we examined the correlation between normalized predicted and target distances.

We observed stronger correlations between ground truth data and the distances predicted by the models trained on non-interpolated data compared to the models trained with imbalanced interpolation when testing on *Dactylorhiza* and *Palaquium* (*R*^2^ = 0.24 compared to *R*^2^ = 0.18 and *R*^2^ = 0.36 compared to *R*^2^ = 0.27, respectively; p < 0.001). In *Lomatium* and *Pterocarpus*, correlations were stronger when training using imbalanced interpolation compared to no interpolation (*R*^2^ = 0.29 compared to *R*^2^ = 0.25 and *R*^2^ = 0.22 compared to *R*^2^ = 0.19, respectively; p < 0.001).

Balanced interpolation yielded higher correlation coefficients compared to imbalanced interpolation when testing on *Dactylorhiza*, *Lomatium*, and *Pterocarpus* (*R*^2^ = 0.21 compared to *R*^2^ = 0.18, *R*^2^ = 0.42 compared to *R*^2^ = 0.29, and *R*^2^ = 0.27 compared to *R*^2^ = 0.22, respectively; p < 0.001). Only in *Palaquium* did imbalanced interpolation outperform balanced interpolation (*R*^2^ = 0.27 compared to *R*^2^ = 0.16; p < 0.001).

## Discussion

Here, we introduce a new DNA augmentation technique, called DNAInterpolator, that generates variations of sequences within a given dataset, guided by the structural context of the original DNA. By recombining information already present in the data, augmentation using DNAInterpolator can improve the performance of deep learning models in taxonomic settings.

**Fig 7:**
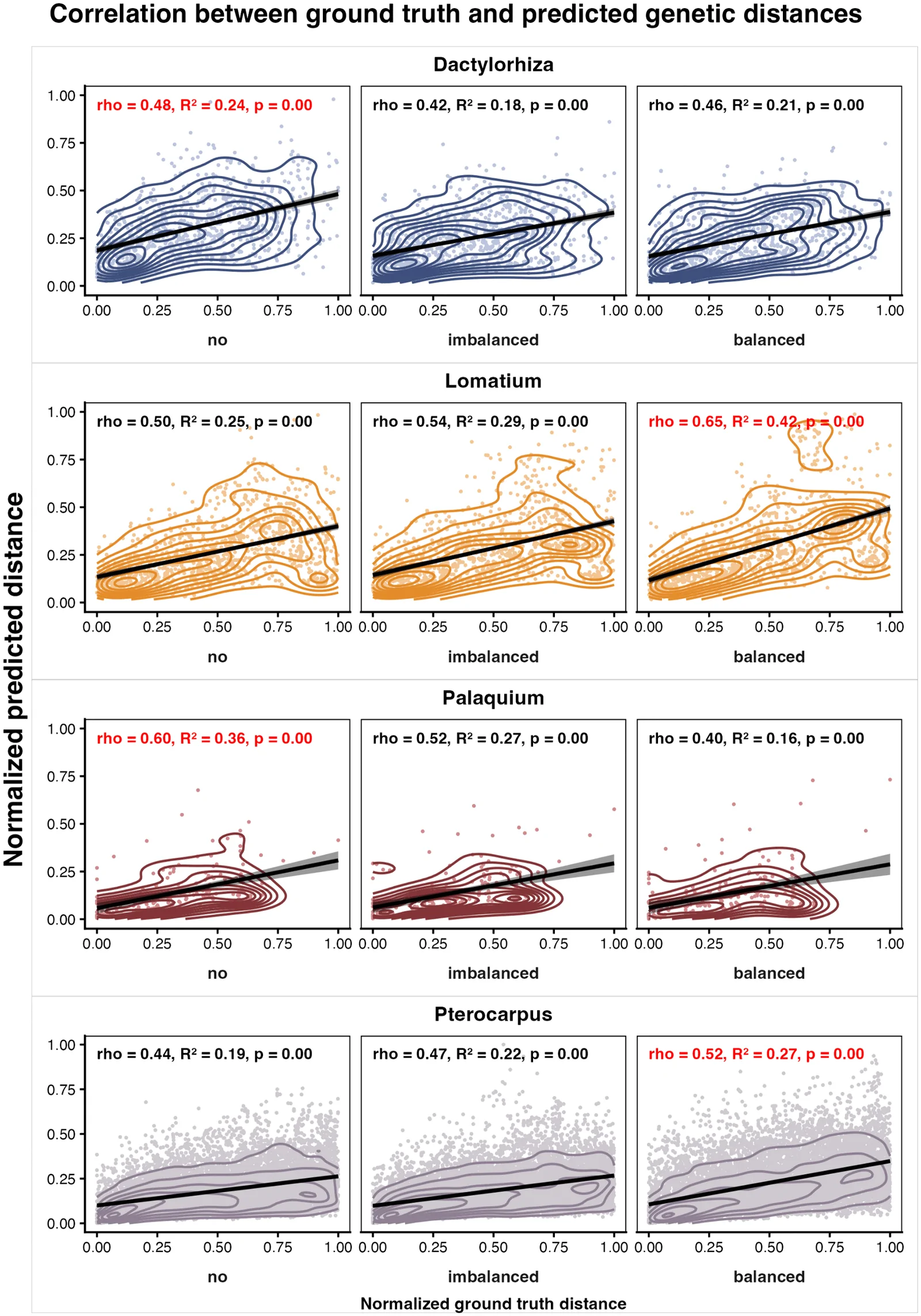
Correlation of predicted and target cosine distances. Visualized are the normalized ground truth distance on the x-axis and the normalized predicted distance on the y-axis for each of the four plant genera and for each interpolation method. Correlation coefficients and p-values were calculated using Spearman’s rank correlation. Red text indicates the best correlation across interpolation methods for the evaluated plant group.

The evaluation of the differences between and the correlation of predicted and ground truth genetic distances show that training on interpolated and balanced data yields more accurate distance predictions than training on non-interpolated data for *Lomatium* and *Pterocarpus*. While not interpolating training data works best for *Dactylorhiza* and *Palaquium*, these findings reflect the overall number of samples within the test datasets, and the biased distance distribution arising from the evolutionary divergence among the Angiosperms353 genes. As shown in Figs 4, 6 and 7, the distance distribution between plant groups varies substantially. Distances between DNA sequences belonging to *Lomatium* and *Pterocarpus* cover the entire range of the cosine distance. With balanced interpolation, the respective distance predictions improved significantly across most of the ground truth distance range, with the exception of sequence pairs below a dataset-specific distance threshold, where the impact was reversed (Fig 6).

Notably, sequence pairs belonging to *Palaquium* do not cover the entire range of the cosine distance. Instead, they are limited to specifically the distance range in which all datasets perform best without any interpolation. The bias in genetic distances is likely due to fast radiations [27,63] and the lowest number of SNPs (~2k) among all tested plant groups.

The bias towards small distances in *Palaquium* leads to a pronounced discrepancy between the training and inference distance distributions. As positive effects of a balanced interpolation approach cannot be observed for very closely related samples (Fig 6), samples belonging to *Palaquium* fall into the small distribution range in which balanced interpolation increases divergence between ground truth and predicted distances. This effect can be attributed to a shifted focus during ML training as the natural distance distribution is distorted and larger distances are more often encountered during training. Consequently, samples with larger distances contribute more frequently to the optimization compared to imbalanced training, leading the model to place greater emphasis on accurately representing long-range relationships. This effect could be mitigated by adjusting the distance cutoff, mapping the cosine distance to a narrower genetic distance range and allowing for a more focused training on the part of the distance range that is most relevant for *Palaquium*.

*Dactylorhiza*, on the other hand, is better suited to test the benefit of either interpolation method due to the substantially larger number of paired samples within the dataset as well as the more balanced distribution of genetic distances. Nevertheless, there remains a minor disproportion between sequence pairs with cosine distances <1 and those with cosine distances >1, with smaller distances being slightly overrepresented in the test set (Fig 4). The bias in genetic distances within *Dactylorhiza* caused by recent polyploid hybrids [30], explains a slightly better correlation coefficient for non-interpolated data than training with interpolated and balanced data. In detail, the relatively small subset of the distance range for which training on non-interpolated data yields consistently more accurate genetic distances has a stronger impact on the overall result due to imbalanced data.

In test datasets such as *Lomatium* and *Pterocarpus* [28,29], which exhibit substantial evolutionary divergence with a high number of SNPs and low levels of reticulate processes, and thus a broad range of genetic distances, balanced interpolation yields the most accurate distance predictions. This observation also represents a limitation of a directed interpolation-based augmentation. The idea behind fine-tuning is to adapt a model to a specific task, narrowing down on those samples that most closely reflect the feature distribution anticipated during inference. When the genetic distances of the inference data deviate significantly from the training and validation data, interpolating and balancing out the distance distribution emphasize parts of the distance range during training that are never encountered at inference time or play a secondary role. In such cases, interpolation does not improve model performance while requiring additional labor and resources depending on the number of samples that should be generated [64]. Therefore, it is advantageous to know the distance range of the test set and to adapt the interpolation and training accordingly.

Our training and test datasets mostly contain closely related genetic sequences. Since DNABERT-2 was trained on a wide range of data from genetically diverse species [12], the cosine distance between the embeddings of very closely related samples is likely to be close to zero. During our training, we therefore aimed to untangle and spread the samples farther apart, forcing them to occupy space across the entire range of the cosine distance. Our results indicate that this process is aided by presenting the model time and time again with what we consider to be distantly related samples (Fig 5), thereby rescaling the spacing of embeddings (Fig 6). By enabling the selection of sequence pairs that fit the desired distance distribution while still ensuring a sufficient number of training samples, augmenting sequences before training through interpolation directly improves genetic distance prediction across most of the cosine distance range.

Population genetic and genomic studies have shown that incomplete or uneven sampling can distort estimates of genetic diversity and subsequent inferences about population structure, particularly among recently diverged groups [65–67]. Similarly, missing taxa or uneven sampling can bias tree topology, branch lengths, and diversification rate estimates in phylogenomic studies with pronounced effects when dealing with rapidly radiating taxa [68,69]. In both contexts, our approach provides a strategy to mitigate sampling-related limitations by generating biologically plausible interpolated sequences that fill sampling gaps when exhaustive sampling is not possible.

DNAInterpolator may also benefit the estimation of a fossil’s genetic identity. In cases where a fossil might only be represented by its morphology, a reliable genetic embedding space could facilitate the linkage between morphological and DNA information. The morpho-genetic mapping could then be used to describe the genetic makeup of the fossil by leveraging the relative position to related taxa. Previous studies used deep learning on morphological data to infer phylogenetic or genetic relationships, either by embedding morphological features into a phylogenetic distance space [70] or by approximating high-level genetic/taxonomic affinities [7], but neither predicts multi-locus, subgenome, or genome-scale DNA embeddings. Our approach would enable the mapping of morphological traits to DNA embeddings for prediction of pairwise genetic distances between fossils and extant taxa. By training with interpolated DNA sequences, we fill gaps within a genetic distance space and improve resolution, particularly among closely related sequences, rare or even extinct taxonomic groups. Furthermore, leveraging multi-locus data across hundreds of genes makes our embeddings far more comprehensive than single-marker studies.

Notably, DNAInterpolator can be applied irrespective of subsequent analyses. Since DNA sequences are interpolated based on their original state rather than their numeric representations, DNAInterpolator does not depend on a specific encoding method such as the regularly used one-hot encoding [37,40]. While we have finetuned DNABERT-2 [12] in this study, we expect balanced interpolation to generalize to other models trained on pairwise similarity relationships, as the method improves coverage and reduces sparsity along the distance distribution. Note, however, that the magnitude of the impact can vary irrespective of the chosen model depending on the degree of overlap between training and inference. Instead of using balanced interpolation, alternative measures can be taken to emphasize the importance of rare targets during training such as introducing special metrics for optimization and evaluation of a model with imbalanced distributions [71]. For researchers, independence from downstream tasks and potential conversion into a numeric format ensure maximum flexibility along with ease of interpretability.

Follow-up developments of our interpolation approach could account for correlations between sites when transferring variants from donor to recipient to further increase the empirical support of the interpolated samples. One possible implementation would be to process codons instead of individual sites. However, the use of codons would require additional annotation and gene trimming. At the same time, introducing further constraints on interpolation may also lead to a more sparsely populated sequence space, impeding the balancing of distances.

DNAInterpolator provides a conservative and transparent approach for sequence augmentation in limited but complex phylogenomic datasets that cover many loci of multiple individuals across species. In contrast to alternative augmentation techniques such as HMMs, DNAInterpolator explicitly preserves within-group (be it species or recipient individual) genetic variation (Fig 2). Group-specific variation is retained by restricting each augmented position to nucleotide states observed at that position in the corresponding dataset and applying explicit limits on deviation from the consensus, e.g., by not introducing additional SNPs. Furthermore, DNAInterpolator records the origin of each selected nucleotide, providing full transparency and control over the augmented data.

In contrast to DNAInterpolator, generative approaches such as HMMs do not only inter- but also extrapolate, expanding the effective sequence space and potentially altering the empirical distribution of pairwise distances. In the context of distance-based model training, this can result in an increased number of sequence pairs exceeding the defined distance range and, consequently, requiring removal, rendering the approach much more ineffective. Alternatively, an expansion of the accepted distance range would result in a disproportionate representation of artificially generated sequences at the upper boundary of the distribution.

Rather than a comprehensive model of sequence evolution, DNAInterpolator is designed as a transparent and conservative augmentation framework that preserves genetic distance structures. It enables model fine-tuning in areas of the distribution that may be underrepresented due to sampling difficulties, rare haplotypes, or other biological constraints by increasing density in sparse sequence spaces. The additional reference points within this denser sequence space can provide crucial information for downstream tasks and stabilize the composition of representations within the embedding space. Ultimately, the aim of DNAInterpolator and genetic-distance-based training is to create sequence representations that capture consistent phylogenomic patterns. Such representations can help assign taxonomic labels, form biologically meaningful clusters, and delimit species with improved interpretability and efficiency.

## Conclusion

In this study, we demonstrated that a selective augmentation of DNA sequences prior to model training can improve the relationships between embeddings within an n-dimensional embedding space to be more biologically meaningful. By interpolating between sequences within the original dataset and systemically choosing samples to balance out the genetic distance distribution observed in the original data, we can reinforce learning of all relationships within a pre-defined distance spectrum. As shown, strengthening of connections within the spectrum decreases distance prediction divergence from the ground truth when the tested samples cover the entire distance spectrum. Interpolating offers another perk in its improved interpretability compared to augmentations introducing context-unaware mutations and indels which are applied during training. In cases where there is an increased interest in the specifics of the data used for training or where there is a need to preserve boundaries between closely related taxonomic groups, our interpolation pipeline provides the necessary control by simulating sequences outside of the model training. Outside of ML, our interpolation approach can alleviate restrictions of limited sampling in phylogenomic studies by providing a biological data-driven simulation of sequences that builds a continuum between original samples.

## Supporting information

S1 Table Training and validation dataset sizes of interpolation approaches. (PDF)

S1 Appendix A–C Figs Supplementary figures showing clustering of GTR+GAMMA distances of interpolated and original *Lomatium*, *Palaquium*, and *Pterocarpus* samples. (PDF)
