# Supplementary material for: Balanced DNA interpolation improves learning of genetic distance-informed embeddings in plants
Source: PLoS Comput Biol. 2026 Sep 3;22(9):e1014722. doi: 10.1371/journal.pcbi.1014722 (PMC13577514; doi:10.1371/journal.pcbi.1014722)
Supplement: Supplementary file 1 [file pcbi.1014722.s001.pdf]

Table S1. Training and validation dataset sizes of interpolation approaches. Sample sizes refer to pairs of sequences with an attached genetic distance.

**No interpolation**					
**LOOCV plant group**	**Training subset**	**Samples**	**Training**	**Validation**	**Validation %**
**All**	*Dactylorhiza*	12,910	9,668	3,242	25.11%
	Lomatium	270,110	207,567	62,543	23.15%
	Palaquium	530,084	420,015	110,069	20.76%
	Pterocarpus	790,003	630,010	159,993	20.25%
Dactylorhiza	Combined	1,590,197	1,257,592	332,605	20.92%
Lomatium	Combined	1,332,997	1,059,693	273,304	20.5%
Palaquium	Combined	1,073,023	847,245	225,778	21.04%
Pterocarpus	Combined	813,104	637,250	175,854	21.63%
**Imbalanced interpolation**					
**LOO plant group**	**Training subset**	**Samples**	**Training**	**Validation**	**Validation %**
**All**	*Dactylorhiza*	633,252	630,010	3,242	0.51%
	Lomatium	692,553	630,010	62,543	9.03%
	Palaquium	530,084	630,010	110,069	14.87%
	Pterocarpus	790,003	630,010	159,993	20.25%
Dactylorhiza	Combined	2,222,635	1,890,030	332,605	14.96%
Lomatium	Combined	2,163,334	1,890,030	273,304	12.63%
Palaquium	Combined	2,115,508	1,890,030	225,778	10.67%
Pterocarpus	Combined	2,065,884	1,890,030	175,854	8.51%
**Balanced interpolation**					
**LOO plant group**	**Training subset**	**Samples**	**Training**	**Validation**	**Validation %**
Dactylorhiza	*Lomatium*	937,629	875,086	62,543	6.67%
	Palaquium	237,881	127,812	110,069	46.28%
	Pterocarpus	1,047,125	887,132	159,993	15.28%
	Combined	2,222,635	1,890,030	332,605	12.96%
Lomatium	*Dactylorhiza*	459,472	456,230	3,242	0.71%
	Palaquium	277,532	167,454	110,069	39.66%
	Pterocarpus	1,426,339	1,266,346	159,993	11.22%
	Combined	2,163,334	1,890,030	273,304	12.63%
Palaquium	*Dactylorhiza*	339,246	336,004	3,242	0.96%
	Lomatium	898,499	835,956	62,543	9.69%
	Pterocarpus	878,063	718,070	159,993	18.22%
	Combined	2,115,508	1,890,030	225,778	10.67%
Pterocarpus	*Dactylorhiza*	1,034,557	1,031,315	3,242	0.31%
	Lomatium	806,226	743,683	62,543	7.76%
	Palaquium	225,101	115,032	110,069	48.9%
	Combined	2,065,884	1,890,030	175,854	8.51%
