# Supplementary material for: Balanced DNA interpolation improves learning of genetic distance-informed embeddings in plants
Source: PLoS Comput Biol. 2026 Sep 3;22(9):e1014722. doi: 10.1371/journal.pcbi.1014722 (PMC13577514; doi:10.1371/journal.pcbi.1014722)
Supplement: Supplementary file 2 [file pcbi.1014722.s002.pdf]

Clustering of individuals

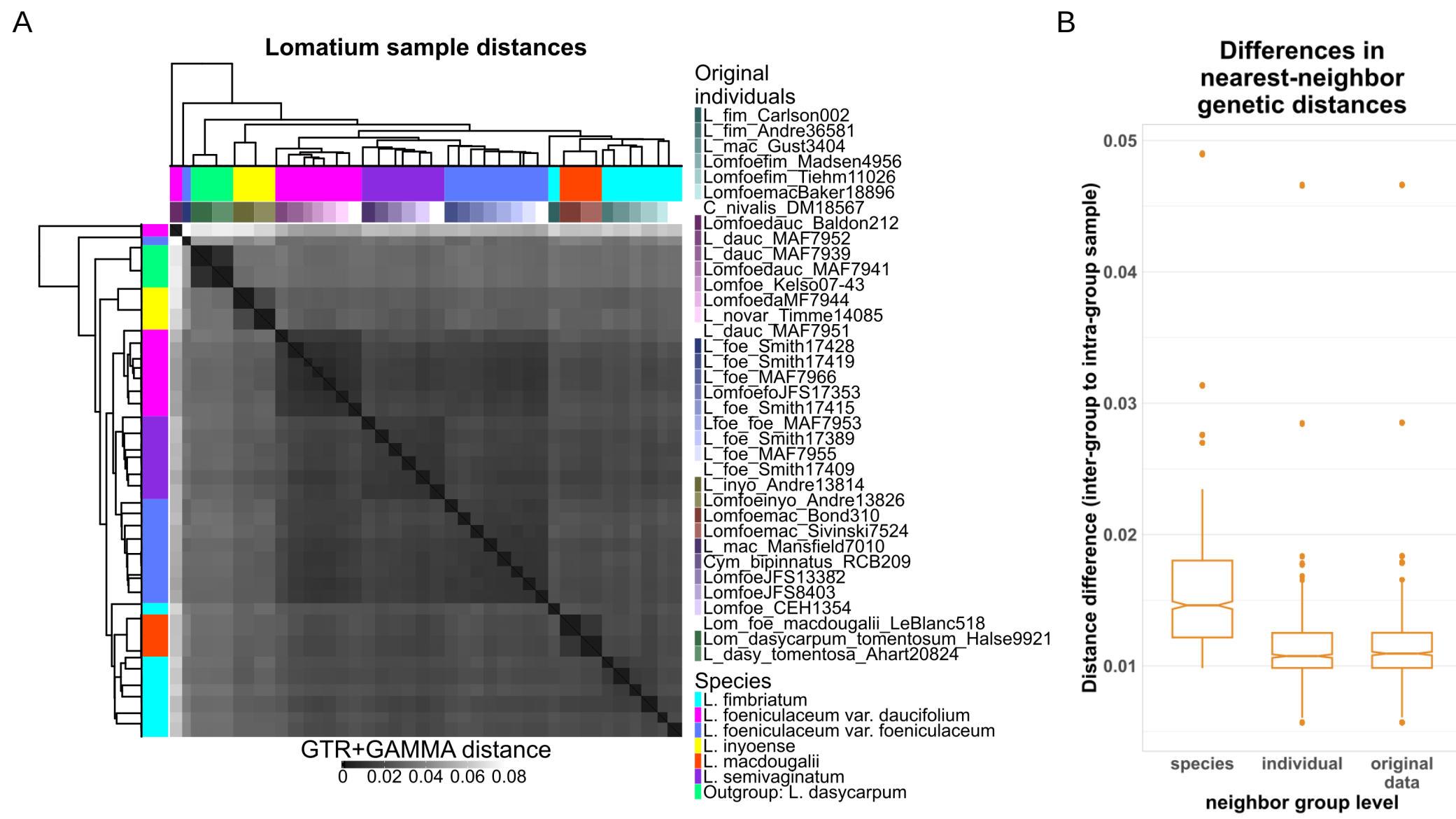

Fig A. Clustering of GTR+GAMMA distances of interpolated and original *Lomatium* samples. A) Shown are GTR+GAMMA distances calculated with RAxML between original and interpolated samples. Each sample is a concatenated sequence of loci that represents an individual. Missing loci were filled with gaps. Colored bars at the top and to the left of the heatmap illustrate species information. The thinner colored bars on top of the heatmap directly below the species information represent original individuals. Interpolated individuals are colored according to the respective original individual (recipient) used during interpolation. B) Shown are differences between genetic distances of nearest neighbors across and within groups. For instance, data points for 'species' include differences between genetic distances of a) individuals belong to the same species, and b) individuals belong to different species. For original data, only genetic distances from interpolated samples to a) the respective original sample, and b) unrelated original samples were considered. Both plots are based on a subset of 1000 original and interpolated samples, where interpolated samples were randomly chosen.

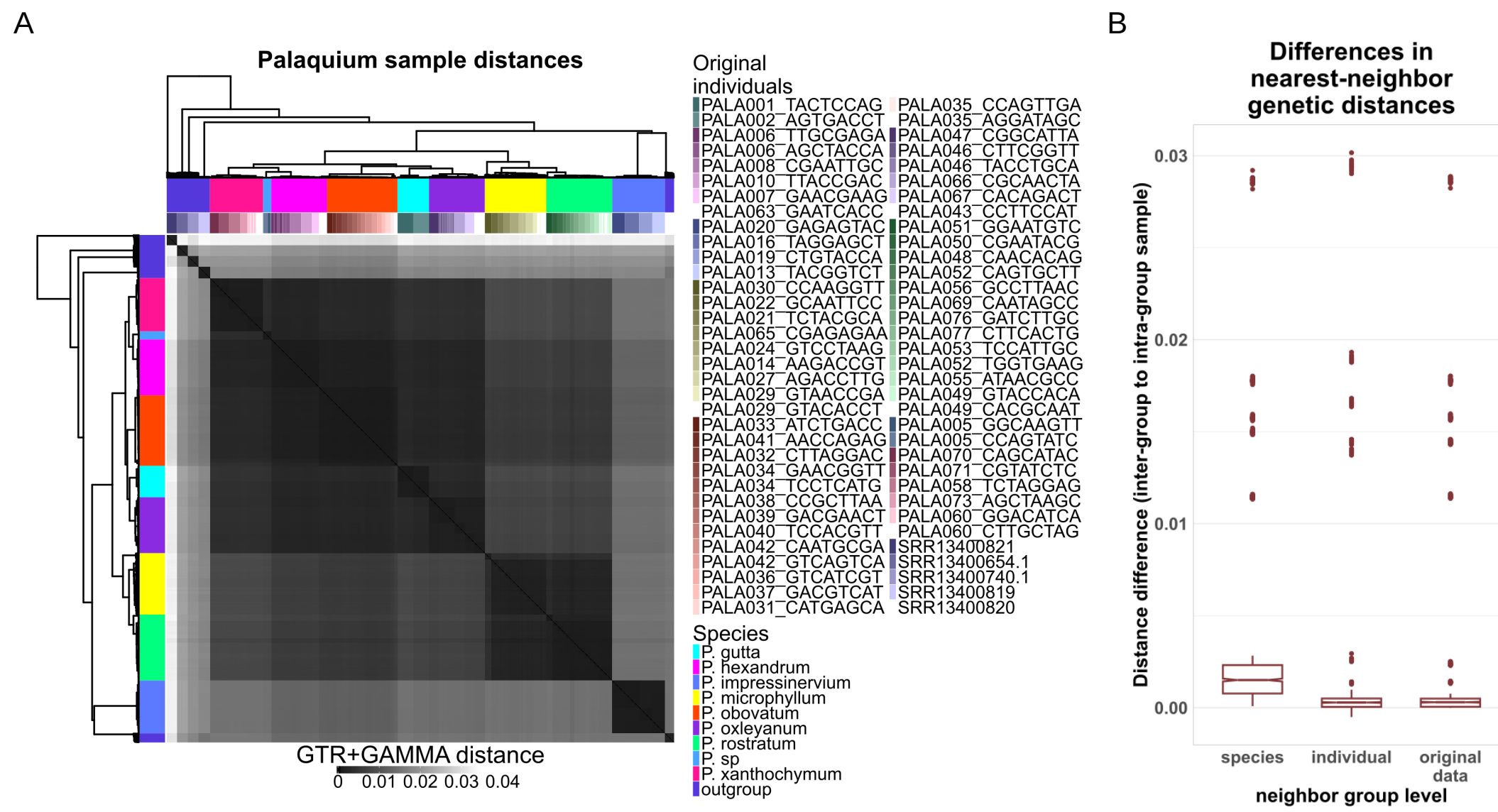

Fig B. Clustering of GTR+GAMMA distances of interpolated and original *Palaquium* samples. A) Shown are GTR+GAMMA distances calculated with RAxML between original and interpolated samples. Each sample is a concatenated sequence of loci that represents an individual. Missing loci were filled with gaps. Colored bars at the top and to the left of the heatmap illustrate species information. The thinner colored bars on top of the heatmap directly below the species information represent original individuals. Interpolated individuals are colored according to the respective original individual (recipient) used during interpolation. B) Shown are differences between genetic distances of nearest neighbors across and within groups. For instance, data points for 'species' include differences between genetic distances of a) individuals belong to the same species, and b) individuals belong to different species. For original data, only genetic distances from interpolated samples to a) the respective original sample, and b) unrelated original samples were considered. Both plots are based on a subset of 1000 original and interpolated samples, where interpolated samples were randomly chosen.

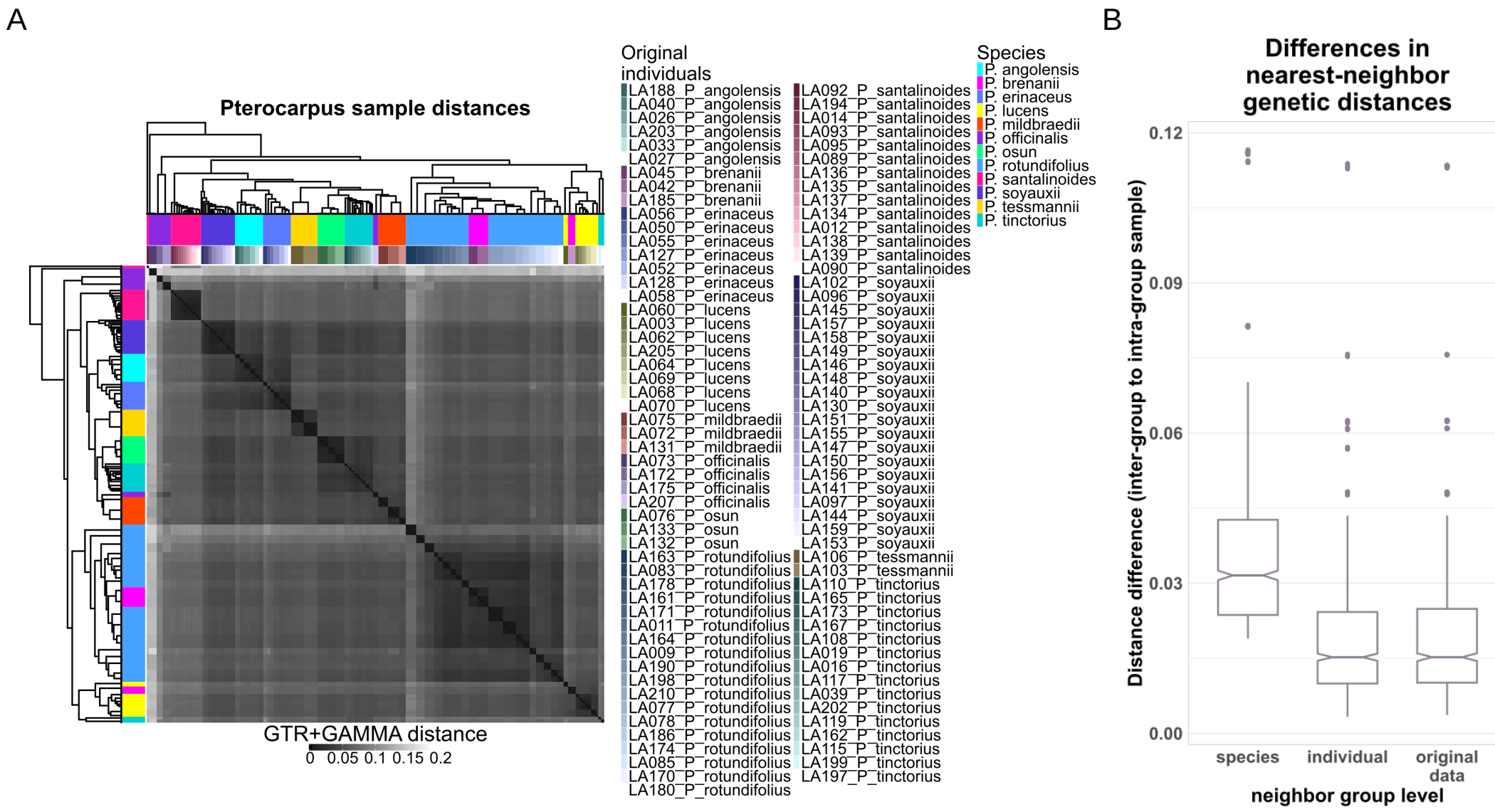

Fig C. Clustering of GTR+GAMMA distances of interpolated and original *Pterocarpus* samples. A) Shown are GTR+GAMMA distances calculated with RAxML between original and interpolated samples. Each sample is a concatenated sequence of loci that represents an individual. Missing loci were filled with gaps. Colored bars at the top and to the left of the heatmap illustrate species information. The thinner colored bars on top of the heatmap directly below the species information represent original individuals. Interpolated individuals are colored according to the respective original individual (recipient) used during interpolation. B) Shown are differences between genetic distances of nearest neighbors across and within groups. For instance, data points for 'species' include differences between genetic distances of a) individuals belong to the same species, and b) individuals belong to different species. For original data, only genetic distances from interpolated samples to a) the respective original sample, and b) unrelated original samples were considered. Both plots are based on a subset of 1000 original and interpolated samples, where interpolated samples were randomly chosen.
